# Complete Genome Sequence of a Novel Porcine Reproductive and Respiratory Syndrome Virus That Emerged in China

**DOI:** 10.1128/genomeA.00702-15

**Published:** 2015-07-09

**Authors:** Feng Zhou, Jun Zhao, Lu Chen, Hong-tao Chang, Yong-tao Li, Hong-ying Liu, Chuan-qing Wang, Xia Yang

**Affiliations:** College of Animal Science and Veterinary Medicine, Henan Agricultural University, Zhengzhou, Henan Province, China

## Abstract

A novel porcine reproductive and respiratory syndrome virus (PRRSV) strain with 393 nucleotide deletions in the nonstructural protein 2 (Nsp2) region was examined in this study. Results will help improve our understanding of the epidemiology and genetic diversity of the North American-type PRRSV in China.

## GENOME ANNOUNCEMENT

Porcine reproductive and respiratory syndrome virus (PRRSV) is an enveloped, positive-sense, single-stranded RNA virus that belongs to the family *Arteriviridae* (order *Nidovirales*); PRRSV has resulted in immense economic losses in the Chinese swine industry since it was first reported in China in 1996 (1, 2). A highly pathogenic PRRSV (HP-PRRSV) with a novel discontinuous 30-amino acid (aa) deletion (i.e., amino acids 481 and 533 to 561 in Nsp2) was first isolated in China in 2006 (3, 4); the European-type PRRSV was reported at the same time (5). The PRRSV epidemiology in China is diverse, although HP-PRRSVs have remained dominant since 2006 (6). Furthermore, PRRSVs have shown remarkable genetic variation through mutation and recombination, thereby resulting in the emergence of many novel variants (7–11). In this study, the complete genome sequence of the novel PRRSV variant HENAN-XINX was examined.

The HENAN-XINX strain was isolated from the lung samples of a piglet that had been vaccinated with a modified live vaccine derived from PRRSV strain VR2332 in a village in Henan province, central China, in March 2013. To determine the complete genome sequence of the HENAN-XINX strain, 10 pairs of specific primers, whose design was based on the sequences of PRRSV strains VR2332 (GenBank accession no. AY150564) and NADC30 (GenBank accession no. JN654459), were used to generate overlapping amplicons by reverse transcription-PCR. In contrast, the 5′ and 3′ regions were acquired through rapid amplification of cDNA ends using a RACE kit (TaKaRa, China). The PCR products were purified, cloned into a pMD18-T vector (TaKaRa), sequenced, and analyzed using DNASTAR (Lasergene) software. The complete PRRSV HENAN-XINX genome was 15,020 nucleotides in length and excluded the poly(A) tail; the genome included the numbers of nucleotides for the following genes: 191 for the 5′ untranslated region (UTR), 11,489 for the Rep gene, 771 for the GP2 gene, 765 for the GP3 gene, 537 for the GP4 gene, 603 for the GP5 gene, 525 for the GP6 gene, 372 for the N gene, and 151 for the 3′ UTR.

Complete HENAN-XINX genomic sequence alignment of HENAN-XINX shared 83.19% and 52.29% nucleotide identity with the American prototype VR2332 and the European prototype Lelystad virus (GenBank accession no. M96262), respectively. HENAN-XINX genomic organization is similar to that of American PRRSV strain NADC30 (GenBank accession no. JN654459) with 95.43% nucleotide identity and the same discontinuous 131-aa deletion at amino acids 303, 323 to 433, and 501 to 519 in Nsp2. This result indicated that the HENAN-XINX strain is a North American-type PRRSV.

HENAN-XINX sequence data indicated that a new PRRSV variant has emerged in China. This information will benefit further research on molecular epidemiology and genetic diversity of PRRSVs in China.

### Nucleotide sequence accession number. 

The complete genome sequence of the HENAN-XINX strain is available in GenBank under the accession number KF611905.
